# Neural Correlates of Auditory Perceptual Awareness under Informational Masking

**DOI:** 10.1371/journal.pbio.0060138

**Published:** 2008-06-10

**Authors:** Alexander Gutschalk, Christophe Micheyl, Andrew J Oxenham

**Affiliations:** 1 Department of Neurology, Ruprecht-Karls-Universität Heidelberg, Heidelberg, Germany; 2 Department of Psychology, University of Minnesota, Minneapolis, Minnesota, United States of America; Newcastle University Medical School, United Kingdom

## Abstract

Our ability to detect target sounds in complex acoustic backgrounds is often limited not by the ear's resolution, but by the brain's information-processing capacity. The neural mechanisms and loci of this “informational masking” are unknown. We combined magnetoencephalography with simultaneous behavioral measures in humans to investigate neural correlates of informational masking and auditory perceptual awareness in the auditory cortex. Cortical responses were sorted according to whether or not target sounds were detected by the listener in a complex, randomly varying multi-tone background known to produce informational masking. Detected target sounds elicited a prominent, long-latency response (50–250 ms), whereas undetected targets did not. In contrast, both detected and undetected targets produced equally robust auditory middle-latency, steady-state responses, presumably from the primary auditory cortex. These findings indicate that neural correlates of auditory awareness in informational masking emerge between early and late stages of processing within the auditory cortex.

## Author Summary

Sounds that are well above the sensory threshold may sometimes fail to be perceived when they occur amid competing sounds, as often happens in everyday life. This phenomenon is generally referred to as “informational masking.” We took advantage of this effect to isolate brain responses that correlate with conscious auditory perception. Human listeners performed an auditory detection task in which they had to indicate when they heard a stream of repeating tones (targets) embedded in a stochastic tone background (masker). At the same time, brain responses were recorded using magnetoencephalography. By comparing the responses to perceptually detected and undetected target tones in the auditory cortex, we isolated a neural response component in the latency range of 50–250 ms, which was only present for detected sounds. We propose that this component, the “awareness related negativity,” specifically reflects conscious sound perception. In contrast, earlier responses in the auditory cortex were evoked by both detected and undetected target tones. These results suggest that conscious sound perception emerges from within the auditory cortex.

## Abstract 3

How does the brain process sound when we can't always be aware of all auditory information at once? A new study shows that auditory processing depends on whether the sound is consciously perceived.

## Introduction

On a busy street corner, in a crowded restaurant, or in a rainforest at twilight, the sounds emitted from multiple sources mix together to form a highly convoluted and complex acoustic environment. Ecologically relevant warning or mating calls, or the speech from your neighbor at a restaurant table, must be heard out of this background cacophony. When a certain sound is not heard out of a background mixture, it is said to be masked. Many examples of masking can be explained in terms of the way sounds are processed in the inner ear, or cochlea [1]. The background or masking sound produces a pattern of excitation in the cochlea that either swamps or suppresses the activity due to the target sound, so that the target is no longer accurately represented in the auditory nerve [2]. This form of masking, traditionally known as “energetic masking,” has been the subject of most formal psychophysical studies of masking dating back nearly 100 years [3]. In general such masking, measured behaviorally, corresponds well to predictions based on physiological measurements from the cochlea or auditory nerve [4,5]. The maskers and targets used in such experiments are typically predictable (i.e., the same sounds are presented over many repetitions), and are easily distinguished from one another.

More recently it has become clear that the principles and predictions of energetic masking may not hold in many natural situations, where competing sounds are neither predictable nor readily distinguishable. Masking under conditions of uncertainty and timbral similarity has been referred to as “informational masking.” The term informational masking, which was initially applied to the perception of elemental sounds, such as pure tones [6,7], has more recently been applied to a wide range of contexts, including the masking of speech by other speech sounds [8]. Although it is unlikely that the same mechanisms underlie all forms of informational masking, they all have in common that the effects cannot be explained in terms of interactions in the auditory periphery (the cochlea and auditory nerve) [9]. In this study, we investigated the neural correlates of informational masking as it applies to the detection of a target tone sequence embedded in a random multi-tone background.

Where and how informational masking occurs in the auditory system remains unknown. In fact, with our current state of knowledge, informational masking may originate at any processing stage along the auditory pathways, from the cochlear nucleus in the brainstem, up to (and possibly beyond) the auditory cortex (AC). We combined a behavioral informational masking paradigm with simultaneous magnetoencephalography (MEG) recordings in humans to investigate the role of the AC in informational masking in particular, and auditory awareness in general.

**Figure 1:**
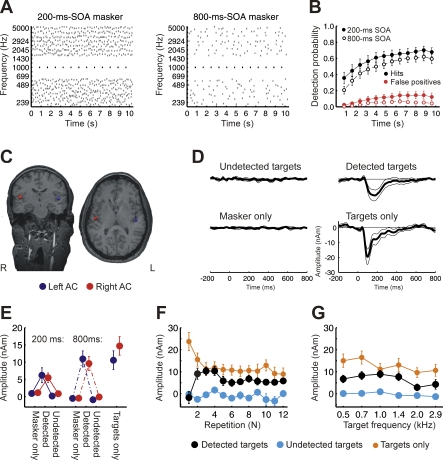
Experiment 1A and 1B (A) Schematic spectrogram of the stimulus paradigm, arranged in 18 log-spaced frequency bands (239–5,000 Hz). The target (black) was a regularly repeating tone (489–2,924 Hz). Two frequency bands above and below the target were kept as protected region; masker tones were present in the remaining frequency bands. The stimulus-onset asynchrony (SOA, interval between the onsets of two subsequent tones) within a masker band was randomized with an average SOA of 200 ms (left panel) or 800 ms (right panel). (B) Average detection probability across listeners (± 1 s.e.m.) for the 200-ms (filled circles) and the 800-ms SOA masker condition (open circles) over time. False positive responses were derived from masker-only conditions. Because the listeners' task was to indicate when they detected repeating target tones, which required that they detected at least two consecutive target tones, the two target tones preceding a detection response (key press) were counted as detected tones in our analysis of behavioral responses. (C) Location of ARN dipoles in the AC for a sample listener. (D) Source waveforms averaged over hemispheres, SOA-conditions, and listeners. Confidence intervals indicate *t*-intervals (*p* < 0.05, two-tailed). As for the behavioral data, the two target tones that preceded a key press were considered detected. (E) Average amplitudes in the time range 75–175 ms after target-tone onset (± 1 s.e.m.), for left (blue) and right (red) AC. The data on the left represent the data from the 200-ms, the middle set the data from the 800-ms masker SOA, and the right-most set the control data with unmasked targets. (F) MEG-source amplitudes for each separate target repetition (mean ± 1 s.e.m., time-range 75–175 ms; average across hemispheres and SOA conditions; *n* = 12). (G) MEG-source amplitudes for each separate target-frequency band (mean ± 1 s.e.m., *n* = 12, except detected targets at 2.0 kHz: *n* = 11).

Our listeners' task was to detect a stream of regularly repeating target tones against a background of masking tones that were randomly placed in time and frequency (Figure 1A). The stimuli are similar to those used in earlier studies of informational masking using random multi-tone backgrounds [10,11], with the exception that our masking tones were not synchronized with the target tones. This desynchronization allowed us to separate the time-locked MEG responses evoked by the target tones from those evoked by the masker tones.

To limit the contribution of energetic masking and peripheral interactions between the targets and maskers, the target tones were separated from the masking tones by a fixed minimum frequency gap or “protected region.” A frequency gap also promotes the perceptual segregation of target and masker tones into distinct sound “streams,” making it easier for listeners to identify the regularly repeating, constant-frequency target tones amid the randomly varying masker tones [12–14]. Although the presence of the target is obvious in the visual representation of Figure 1A, the targets in this configuration were not clearly audible; in fact, listeners reported hearing them on only about half the presentations. On some trials, the target tones “popped out” from the background and became clearly audible well before the end of the stimulus sequence; on other trials they were not heard at all. Such dramatic changes in perception from one trial to the next are typical in informational masking experiments. Because detection in this task is not associated with systematic changes in the physical stimuli (the exact same stimulus can elicit detection on one occasion and not on another), this paradigm provides ideal conditions for identifying neural correlates of auditory awareness, independent of both physical stimulus manipulations and peripheral auditory interactions.

We compared MEG signals that were time-locked to either detected or undetected target tones in the AC. We identified robust early AC responses (the middle-latency steady-state responses—SSR) to the target, which remained the same whether the target was detected or not. Changes in later AC responses, starting approximately 70 ms after target onset, were found to depend critically on whether listeners were aware of the target tones. This longer-latency MEG response was strong when listeners reported hearing the target, but was not measurable when listeners failed to detect the target, or when their attention was directed elsewhere. The finding of robust early neural responses in the AC to sounds, regardless of whether they are detected, in conjunction with later AC responses that are highly correlated with detection, suggests that auditory awareness in a classical informational masking paradigm emerges from within the AC, rather than in lower-level brainstem or higher-level supra-modal cortical structures.

## Results

### Experiment 1A and 1B: Behavior

In the first experiment, listeners were presented with 10.4-s stochastic tone sequences generated by adding multiple tone bursts with pseudo-random frequencies and onset times. In two-thirds of these random-onset multi-tone sequences, a tone repeating regularly at a constant frequency throughout the sequence was added (Figure 1A). To indicate when they were aware of these targets, listeners were instructed to press a key as soon as they began to hear the regularly repeating target tones against the randomly varying background tones. The probability that listeners detected the target stream increased over the duration of each sequence, reaching on average about 0.6 by the end of the sequence. The rate of false-alarms (i.e., target-detection responses on trials in which only the masker tones were presented) also increased slightly over the course of the stimulus sequence, reflecting listeners' increasing expectation to hear out the target tones, but remained low overall (Figure 1B). The listeners' unbiased detection performance, *d'*, computed as the difference between the *z*-transformed hit and false-alarm rates, increased over the duration of the sequence, reaching an average value close to 2 at the end (Figure S1).

The experiment was repeated with two different informational maskers: in one (Experiment 1A), the average stimulus-onset asynchrony (SOA), defined as the time interval between the onsets of two consecutive tones within each of the masker frequency bands, was 200 ms; in the other (Experiment 1B), the average SOA was 800 ms, producing a more sparsely populated masking stimulus (compare left and right panels in Figure 1A). The behavioral results obtained with these two variants of the experiment were very similar overall (Figure 1B). Although hit and false-alarm rates were slightly higher in the 200-ms SOA condition than in the 800-ms SOA condition, the average values of *d'* (1.81 and 1.76, respectively) did not differ significantly from each other (*F*_(1,11)_ = 0.09; *p* = 0.7674; Figure S1), indicating that the amount of informational masking was essentially the same in both conditions. Therefore, the data from Experiments 1A and 1B were pooled in most instances for the analyses presented below.

### Experiment 1A and 1B: Long-Latency Responses to Detected and Undetected Targets

To determine whether time-locked brain activity in response to the targets depended on them being consciously detected by the listeners, MEG responses to detected target tones were averaged separately from MEG responses to undetected target tones. Detected targets evoked a prominent bilateral wave with maximal amplitudes on gradiometers positioned over the temporal lobes in the time range from 50 to 250 ms after stimulus onset. The topography of this wave was similar to that of the well-known N_1_m, evoked by single tones in silence. A source analysis with two dipoles, one for each auditory cortex, consistently resulted in dipole locations in Heschl's gyrus or planum temporale, or very close to it, with respect to the listeners' individual magnetic resonance imaging (MRI) anatomy (Figure 1C). Averaged across listeners, Talairach coordinates (Table 1) were located in the central AC, at the border between Heschl's gyrus and planum temporale, as determined in representative populations [15,16]. The variance was similar to that found for other components generated in the auditory cortex [17,18]. The location of fitted dipoles in the presence of the masking tones was not significantly different from the location of the dipoles fitted to the N_1_m measured in the target-alone condition, in the absence of any masking tones (*F*_(2,22)_ = 0.028; *p* = 0.7603).

**Table 1:**
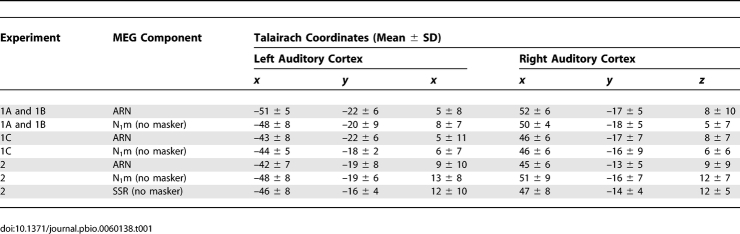
Mean Dipole Locations ± Standard Deviations (*n* = 12) in the Space of Talairach and Tournoux (1988)

The fitted dipoles were then used as a spatial filter to generate source waveforms [19], estimating the time course of MEG activity in the auditory cortex. The source waveforms were qualitatively very similar when the detected-target or the target-only conditions were used to fit the dipoles, and the following summary is based on the detected-target conditions. The source waveforms associated with these dipoles are shown in Figure 1D (confidence intervals represent bootstrap based *t*-intervals, *p* < 0.05, two-tailed).

The averaged response to detected target tones showed a prominent negativity (detected versus undetected targets: *F*_(1,11)_ = 32.15; *p* = 0.0001), peaking around 120–200 ms after tone onset [mean peak latency: 183 ± 14 ms s.e.m. (200-ms-SOA masker); 141 ± 9 ms s.e.m. (800-ms-SOA masker)]. The wave was broad-based, and the deviation of the trace from 0 was statistically significant (*p* < 0.05) everywhere in a 71–283-ms range around the negative peak. There was no significant difference in amplitude (*F*_(1,11)_ = 0.63; *p* = 0.4425) or latency (*F*_(1,11)_ = 1.16; *p* = 0.3044) between right and left hemispheres. We refer to the negative wave evoked by the detected tones as the “awareness related negativity” or ARN. This functional label was chosen for convenience and to avoid premature assignment to another response component; it does not imply that the ARN is necessarily a completely separate component of the auditory evoked fields. The ARN peak was somewhat smaller in magnitude and longer in latency than the typical N_1_m evoked by the target tones presented without the masker (lower right panel in Figure 1C), which peaked at 108 ms (±10 ms s.e.m), and was significant from 50 to 276 ms post stimulus onset (*p* < 0.05).

In contrast, the average MEG response to undetected target tones was essentially flat, similar to the average response to sequences containing only the masker and no target (undetected targets versus masker-only trials: *F*_(1,11)_ = 4.12; *p* = 0.0672). The fact that the no-target trace is flat confirms our expectation that the systematic randomization of masking-tone onset effectively cancelled out the responses to the masking tones. In addition, this trace provides a baseline against which the responses to undetected targets can be compared. The results suggest that the responses to undetected targets are very similar to those found for no targets.

#### Potential effects of target order and frequency.

The behavioral data (Figure 1B) show that target tones were more likely to be detected when they occurred near the end of the sequence (linear-contrast analysis: *F*_(1,11)_ = 79.13; *p* = 0.0001). If the ARN is related to listeners' awareness, it should remain present throughout a sequence of detected targets, whereas undetected targets should not evoke an ARN during any part of the sequence.

The time-dependence of our behavioral data suggests another possible interpretation of the ARN, if it is assumed that the amplitude of the ARN actually increases over the duration of the sequence: it may be that the increasing probability of detection over time co-varies with increasing evoked amplitude of the ARN over time, producing a spurious correlation between ARN amplitude and detection. This alternative seems unlikely, given that the undetected-target conditions produced no measurable ARN. Nevertheless, we tested this possibility further by separately computing the magnitude of the response to detected and undetected targets for each target-tone position in the sequence. The results of this analysis are shown in Figure 1F. The mean magnitude of the response to target tones alone (in the absence of masker tones) is shown for comparison. As can be seen, a marked difference between the neural responses to detected and undetected targets was present for all but the first target in the sequence in the time range 75–175 ms (two tailed *t*-tests; *p* < 0.05).

**Figure 2:**
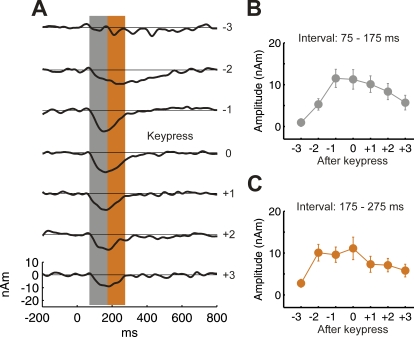
ARN in Relation to Behavioral Response Time (A) ARN source waveforms of experiment 1 (A and B), averaged relative to the behavioral response of the listeners (key press) instead of their position in the stimulus sequence. A strong negativity is evoked by the two tones prior to the key press. The negativity is delayed and more broad-based two tones before the key press. Accordingly, the negativity is smaller in the time interval from (B) 75–175 ms (used for most analyses throughout the paper) than in the time interval (C) 175–275 ms. The data in panels B and C represent the mean amplitude and standard errors across listeners (*n* = 12).

Since the listeners' task was to detect *repeating* target tones, which required that they detected at least two target tones, the two target tones preceding a detection response (key press) were counted as detected in both the behavioral and MEG data analyses. To investigate the temporal relationship between overt detection (as indicated by the key press) and the time course of ARN appearance, the responses in target-detected conditions were realigned and averaged based on the timing of the listeners' key presses. The results confirmed significant negativity evoked by two tones before the key-press, although the response evoked two tones prior to key-presses was smaller, and delayed in latency compared to the waves evoked by all subsequent tones (Figure 2). After the key-press, the ARN shows some adaptation (Figure 2), similar to the N_1_m evoked in the target-only condition. The delay observed for two tones before the key press is reflected by the higher amplitude in the time interval 175–275 ms, compared to the time interval 75–175 ms used for analysis of the ARN throughout this paper (Figure 2C). In fact, when the analysis shown in Figure 1F was done for the 175–275-ms time interval, a significantly stronger negativity for the detected compared to the undetected targets was observed for all target tones in the sequence, including the first (*t* = 3.1, *p* = 0.0100).

Another possible source of averaging bias relates to the finding that targets in the higher-frequency bands were detected less frequently than in lower-frequency bands (*F*_(5,55)_ = 8.68; *p* = 0.0002). Again, however, the magnitude of the response to the target tones was consistently larger for the detected than the undetected target tones, even when the analysis was carried out separately for each of the six target frequencies (Figure 1G; planned comparisons using two tailed *t*-test; *p* < 0.05). Thus, the ARN is not due merely to averaging the responses to targets that occupy different temporal or spectral positions.

### Experiment 1C: Modulating Informational Masking without an Active Task

In experiment 1A and 1B, recording of the ARN was coupled to an active task that involved motor responses. Here we evaluated if the ARN could also be recorded when no active task was performed while listening. Subjects listened passively to a set of shorter-duration sequences (4.8 s), consisting of six target tones and an 800-ms SOA random-onset multitone masker, as well as control conditions comprising only target or only masker tones. Because the listeners' perception remained unknown in the passive setup, an additional manipulation was introduced: the identical masker and target sequences were presented twice (at random positions within the presentation), once in isolation, and once preceded by a cue before the target, in an effort to make the subsequent target tones more detectable [20]. The cue consisted of three tones of identical frequency, and presented with the same 800-ms SOA, as the subsequent target tones, which were presented in silence prior to the multitone masker. It was assumed that the ARN would be larger in the cued condition, because of reduced uncertainty and informational masking, allowing for a larger number of consciously perceived target tones in the cued trials.

**Figure 3:**
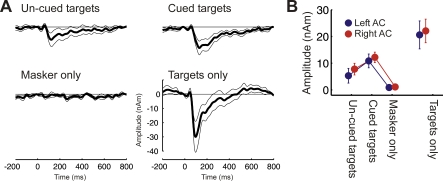
Experiment 1C: Cued Targets In this experiment, listeners attended passively to the stimulation. Targets started either after the informational masker (uncued targets) or two tones before the informational masker (cued targets). Except for the cue, the masker and targets used in the two conditions were identical. This setting was chosen to test the influence of perception on the ARN without the necessity for an active task by the listener. Listeners were naive as to the experiment's objective. (A) Average source waveforms across listeners (*n* = 12) and hemispheres. Confidence intervals represent bootstrap-based *t*-intervals (two-tailed, *p* < 0.05). (B) Mean amplitudes and standard errors of the mean in the time window from 75–175 ms post target tone onset. The ARN evoked by cued targets is significantly stronger than the ARN elicited by uncued targets.

The results of experiment 1C confirmed this prediction (Figure 3). A significantly larger ARN was evoked by the six target tones following the cue compared to the six non-cued target tones (*F*_(1,11)_ = 15.67; *p* = 0.0022; difference significant from 57 to 307 ms, *p* < 0.05). This result indicates that the ARN does not depend on motor preparation or other task-related processes.

### Experiment 2: Attentional Influences on Long-Latency Responses

Conscious detection of a target tone will generally be expected to involve the allocation of attention toward the target. In the context of a multitone masker (without a cue), the perceptual salience of the target is comparatively low, and the contribution of bottom-up (exogenous) mechanisms that could attract attention toward the targets is unlikely to be very strong. Therefore, the direction of attention toward the targets is likely to facilitate detection, while directing attention away from the targets is likely to impair detection. If so, directing listeners' attention away from the targets should lead to a reduced or absent ARN in response to the targets. In experiment 2, the target and masker tone sequences were presented to the left ear only, while an unrelated stimulus sequence, containing occasional “deviant” tones interspersed among standard tones, was presented to the right ear (see Materials and Methods for details). In the first phase of this experiment, the listeners were instructed to detect the deviant tones in the right ear. They were not informed that regularly repeating tones would sometimes be presented to the left ear and, when later interviewed, ten listeners reported that they had heard only irregular bleeps in their left ear; only two listeners reported occasionally noticing regularly repeating tones. In the second phase, listeners were instructed to attend to stimuli in the left ear, ignoring tones in the right ear, and to indicate when they detected the regularly repeating target tones. The stimuli used in the two phases of the experiment were identical.

**Figure 4:**
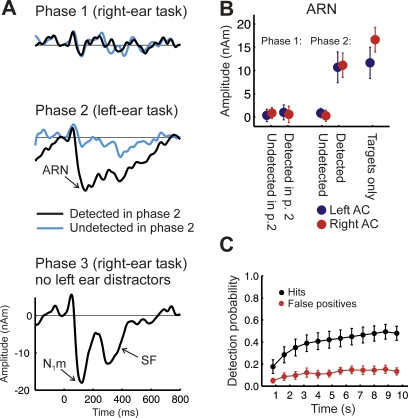
Experiment 2: ARN (A) Grand average activity evoked by targets presented to the left ear (masker-only condition subtracted). The middle panel shows the data from the second phase of the experiment, where subjects listened to the stimuli presented to their left ear, and indicated whether they did (black wave) or did not (blue wave) hear the target stream. The ARN is observed when listeners indicated conscious perception of the target stream. The top panel shows an average over the same selection of physically identical trials (based on the responses in phase 2), when listeners performed the right ear target detection task in phase 1. This condition was recorded first, and listeners were generally not aware of the target stream. The bottom panel shows the activity evoked by an unmasked target stream on the left ear, while subjects were attending to the right. The N_1_m and sustained field (SF) are now evoked without active attention. (B) Mean source amplitudes (± 1 s.e.m.) in the time window 75–175 ms post target tone onset; dipoles were fitted to the ARN. (C) Behavioral data during the left ear task in phase 2.

The average percentage of correct responses in the right-ear deviant-detection task (first phase of the experiment) was 86.8% (±11.7%, S.D.) and the percentage of false alarms was 2.1% (±2.7%, S.D.), yielding a *d'* of 3.4 (±0.7 S.D.). This high level of performance confirms that listeners were attending to the right-ear sequence, as intended. The MEG responses to the target tones were averaged into two groups, depending on whether those same (identical) physical stimuli were detected or undetected in the second phase of the experiment (see below). The MEG responses collected during this first phase (top panel in Figure 4A) reveal that no ARN was evoked by target tones in the left ear when attention was directed away from them (all targets versus masker epochs: *F*_(2,22)_ = 0.23; *p* = 0.7970). The traces were similar to those obtained during epochs where listeners had not detected the targets in the second phase of the experiment, where they were attending to the targets, comprising a P_1_m and a hint of an N_1_m.

In the second phase of the experiment, where the task was to detect the repeating target tones in the left ear (as in the original experiment), the average percentage of correct detections (Figure 4C) was 40.2% (±24.5 S.D.), corresponding to a *d'* of 1.05 (Figure S1B). The MEG responses collected during this second phase (middle panel of Figure 4A) confirm the findings of the first experiment. They show a clear ARN in response to detected targets. No ARN was observed in the average MEG response to undetected targets (detected versus undetected targets: *F*_(1,11)_ = 25.93; *p* = 0.0003; no significant hemisphere effects; undetected targets versus masker-only epochs: *F*_(1,11)_ = 1.24; *p* = 0.2900).

In a third and final phase of this experiment, the regularly repeating target tones were presented in the left ear without the multitone masker, while listeners again performed the right-ear distraction task. Although performance was similar to that measured during the first phase [correct responses: 82.4% ± 11.5%; false alarms: 1.6% ± 2.0%; *d'* = 3.3 ± 0.8 (mean ± S.D.)], listeners now reported being aware of the presence of regularly repeating tones in their left ear. Likewise, the unattended left-ear targets evoked a prominent N_1_m (bottom panel in Figure 4A), and a short sustained field because of the longer tone duration (250 ms versus 100 ms; note that the ARN was also more sustained). Dipole locations for the N_1_m were not significantly different from those for the ARN measured in the second phase of the experiment (*F*_(2,22)_ = 0.64; *p* = 0.5264), pointing to a generator of the ARN and N_1_m in the auditory cortex (Table 1).

### Experiment 2: Auditory SSR to Detected and Undetected Targets

If informational masking were completely pre-cortical, neural correlates of target detection should be readily observed in the earliest cortical responses. To address this prediction in experiment 2, we added sinusoidal amplitude modulation (AM) to the target tones in the left ear at a rate of 40 Hz. This allowed us to selectively record the middle-latency SSR evoked by the target tones, which has been identified in earlier studies as an index of early processing in the auditory core region of Heschl's gyrus [21–23].

**Figure 5:**
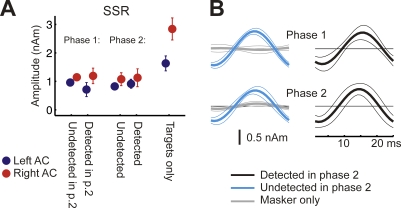
Experiment 2: SSR (A) Mean source amplitudes (± 1 s.e.m.) calculated from peak to peak for the SSR. (B) Grand average source waveforms of the SSR elicited by the amplitude-modulated targets in the presence of the masker. Confidence intervals represent bootstrap based *t*-intervals. The SSR was evoked irrespective of target-tone awareness and side of attention.

Compared to the ARN results reported above, fundamentally different findings were obtained for the SSR evoked by the 40-Hz AM of the target tones (Figure 5). First, the SSR was present in both phases of the experiment, regardless of which ear the listener was attending. Second, when the listeners were attending to the target tones (second phase of the experiment), the SSR was observed regardless of whether or not the target tones were detected. The lack of significant SSR in the masker-only condition (gray waves in Figure 5B) confirms the specificity of this measure for the target tones. The masker-evoked SSR was successfully canceled out by the averaging procedure, because the AM frequencies and onset phases of the masker tones were randomized. Overall, the SSR in response to the target tones was not significantly affected by either target detection (*F*_(1,11)_ = 0.14; *p* = 0.7125) or attention (*F*_(1,11)_ = 0.02; *p* = 0.9008). There were no significant hemisphere effects in the presence of multitone masking, but the SSR was larger in the contralateral (right) hemisphere for AM tones presented in silence (*F*_(1,11)_ = 22.57; *p* = 0.0006).

Using the SSR, we detected no differences in early processing of detected and undetected target tones in the AC. This negative finding does not exclude the possibility of differential early processing of detected and undetected targets by mechanisms in or before the AC that are not reflected in this particular analysis. Nevertheless, the SSR data do show that the target tones are represented in the AC, even when they are not consciously perceived by the listener.

## Discussion

### The Role of the AC in Perceptual Awareness

The present results demonstrate a clear co-variation between late neural responses from the human AC and listeners' awareness of sounds presented well above their detection threshold in quiet, and not masked in the sensory periphery. At the same time, the results demonstrate earlier neural responses in the AC to tones that remain undetected by the listener.

The two MEG components studied here, the SSR and the long latency ARN, are both generated in the AC but reflect different processing stages. Conventional averaging of the SSR was used to maximize early phase-locked activity, and suppress later and non–phase-locked gamma-band activity [24,25], to ensure that the SSR was specifically evoked by the target tones. The phase-locked SSR is tonotopically organized [21,26], and is related to the middle-latency (20–50 ms) response [22,23,27], which, like the SSR, is mainly generated in the auditory core area [21,23,24,28,29]. Thus, the presence of the SSR during undetected tones provides a dissociation between the early activity in the AC and perceptual awareness, suggesting that although early activity in the auditory core may be necessary for perceptual awareness [30], it is not sufficient [31].

In contrast to the SSR, the ARN appears to be closely related to the listeners' perceptual awareness of the target tones, as it was not observed for undetected or unattended targets. Source analyses performed on these data clearly indicate that the ARN is generated in the AC, around Heschl's gyrus. However, the dipole source analysis does not permit us to estimate the extent of the ARN source. Based on its latency and polarity, the ARN might be related to the auditory evoked N_1_m and Nd components. In contrast to the SSR, these components have been shown to be generated across multiple fields of the AC, including lateral Heschl's gyrus, planum temporale, and the superior temporal gyrus [17–19,32–34], comprising the secondary or “belt” regions of the AC [35,36].

In summary, the present data indicate that the neural correlates of auditory perceptual awareness, as measured in the context of a relatively simple informational masking paradigm, can be found between early and late processing stages in the AC. In a finer anatomical view, these processes might be situated in core and belt areas of the AC [35,36], respectively, although there is only indirect evidence for the latter hypothesis at present.

### Previous Electroencephalography Studies of Masking and Detection

In comparing the present findings to those of earlier studies, it is important to distinguish between the two forms of masking—“energetic masking” and “informational masking”—outlined in the introduction. Earlier studies have shown that auditory evoked electroencephalography (EEG) and MEG responses, including subcortical as well as cortical responses, can be strongly attenuated or abolished by the addition of masking noise [37–39]. The type of masking used in these studies corresponds to energetic masking, involving noise that overlaps in frequency and time with the target, which is commonly thought to originate at a peripheral level, reflecting direct physical interactions between the signal and the masking noise within the cochlea [2]. Using energetic masking and selective averaging based on listeners' responses, previous EEG studies have shown that waves P_3_ and N_1_ were observed over the vertex for detected targets only [40,41]. The P_3_ is currently thought to reflect activity in frontal and parietal cortex [42], usually related to active task performance and novelty detection [43]. The AC might have additionally contributed to the N_1_ observed in one study [41], but this was not investigated.

In contrast to these earlier findings, the present results cannot be explained in terms of peripheral interactions between signal and masker, or in terms of novelty-detection or task-performance effects. First, the use of a protected spectral region around the target tones greatly reduced the influence of peripheral interactions between signal and masker. Second, the use of stimulus sequences containing multiple tone bursts, combined with a task that required listeners to report only the first detected target-tone repetition in an ongoing stream, dissociated perceptual detection from task-performance, and novelty effects. Finally, our finding that the ARN can be modulated by cueing listeners to the target tones, even when they were not actively performing the detection task, rules out an explanation in terms of task-performance effects.

### Possible Mechanisms in the AC Related to Informational Masking

The finding of early cortical activity that is independent of detection on the one hand, and of a strong relationship between the longer-latency ARN and listeners' detection on the other hand, strongly suggests a neural correlate of detection within the AC for the multi-tone informational masking paradigm used here.

A number of processes within the AC may determine whether a target is subject to informational multi-tone masking or not. One factor likely to play an important role is selective attention. The ARN had a similar source location to that of the N_1_m, which is evoked by target tones in the absence of the masker, and the two responses largely overlapped in time. The N_1_m has traditionally been considered an “automatic” component, which does not critically depend on overt attention [44]. However, this view is based mostly on results obtained under very low attentional loads, where the sounds evoking the N_1_m were not accompanied by other, competing sounds (as in the target-only control in experiment 1). In experiments with higher processing loads, where multiple sound streams are present, selective attention has been found to modulate responses in the AC [33,34,45–47]. However, a salient N_1_m is still observed in such settings (as in the target-only control in experiment 2), and listeners are usually aware of the presence of the unattended sound stream. It seems that only at very high processing loads, such as under the informational masking paradigm used here, is this response suppressed to the point where it is not measurable if the target is unattended or remains otherwise undetected.

Taking our results together with those of earlier studies, we suggest that the degree to which selective attention affects later AC activity (like the N_1_m) may be explained by attentional load, with higher load leading to greater attentional modulation of the evoked responses. This explanation seems consistent with findings in the visual system, where selective attention has been shown to influence the competition for neural representation in cortex [48,49].

In our experiments, listeners were not able to attend selectively to the target tones from the beginning of each sequence, because the frequency at which the target tones were presented differed across presentations. Recent work has shown that under such circumstances, the detection of the target tones nonetheless occurs more rapidly than predicted by a serial search model, indicating additional bottom-up processes, such as an auditory “pop-out” effect [14]. This pop-out effect is expected to be closely related to automatic auditory-scene-analysis mechanisms, which are thought to parse acoustic stimuli based on low-level features (such as frequency distance, temporal proximity, or spectral continuity over time) and contribute to the formation of auditory streams [11,50]. Recent studies have identified neural phenomena that might subserve the formation of auditory streams in the AC [51–54], and these streaming mechanisms may then again interact with mechanisms of selective attention via bottom-up activation of the ventral fronto-parietal attention system [55].

Based on these considerations, we suggest that, subsequent to early activation of the auditory core, limited processing resources in the AC [56] are a cause of informational masking, once a certain processing load is exceeded. Bottom-up mechanisms subserving stream segregation [11,50], on the one hand, and top-down mechanisms of selective attention [55], on the other hand, may bias the competition between auditory streams. This in turn may help determine the processing resources allocated to different streams within the AC, starting after 50–70 ms, in a manner that appears to be critical for auditory perceptual awareness.

## Materials and Methods

### Listeners.

Thirty-three listeners without history of hearing disorders participated in the study. Three groups of 12 listeners each (six male, six female) participated in experiments 1A and 1B (one group), 1C, and 2. One listener participated in all three experiments, and another one in all parts of experiment 1; the other listeners were different in each experiment. The study protocol was approved by the institutional review board of the University of Heidelberg Medical School; all participants provided written informed consent.

### Stimuli and procedure.

*Experiment 1*: All stimuli were generated using a set of 18 frequency bands, whose center frequencies were spaced equally on a logarithmic scale between 239 and 5,000 Hz (239, 286, 342, 409, **489**, 585, **699**, 836, **1,000**, 1,196, **1,430**, 1,710, **2,045**, 2,445, **2,924**, 3,497, 4,181, and 5,000 Hz). The target tones were selected from the six frequencies shown in bold, and remained constant throughout a 10.4-s sequence. Target tones were 100 ms in duration, including 10-ms on and off cosine-shaped ramps, and were repeated 12 times with a constant SOA of 800 ms.

Two frequency bands on either side of the target frequency were excluded, as a “protected region,” such that the masker comprised the remaining 13 frequency bands. Within each frequency band, the masker-tone frequency was chosen randomly around the center frequency (*f*_c_) within the width of one estimated equivalent rectangular bandwidths [ERB = 24.7 × (4.37 × *f*_ c_ + 1)], where *f*_c_ is in kHz [1]. The masker started 800 ms before the target, resulting in a 10.4-s total duration for the sequence. The SOA between tones was randomized in the range of 100–300 ms or 100–1,500 ms, yielding average SOAs of 200 ms (experiment 1A) or 800 ms (experiments 1B and 1C; the tone density and overall masker energy was accordingly lower in this case).

Each of the six target frequencies was presented together with ten differently randomized masker sequences. Five of the ten masker sequences were also presented without the target tones. The resulting 90 different sequences were presented in random order, separated by silent intervals of 1.6 s. Five repetitions of the targets alone (without the masker) were presented as a control condition at the end of the session. All tone sequences were presented diotically (to both ears). The level of the target tones was 40 dB sensation level (SL) per tone, and the level per tone of the masker was set 18 dB higher.

In experiments 1A and 1B, listeners were familiarized with the stimuli before MEG recordings, and they were informed that the regularly repeating tones would not always be present (although they were not told on what proportion of trials, or whether they would start and end at the same or different times). They were instructed to press the left button of the computer mouse whenever, and as soon as, they detected the repeating target tones. Listeners were encouraged to respond as quickly as possible after the onset of a new sequence, and they were told to press the right mouse button if the sequence ended before the masker, or if they had pressed the left button in error.

In experiment 1C, listeners were instructed to listen passively to four types of stimulus sequences presented in random order. The first three were similar to the conditions of experiment 1B, but comprised only six consecutive target tones (yielding a total duration of 4.8 s). The fourth type of stimulus sequence was obtained by adding three target tones in front of the original target sequence. The unmasked tones at the beginning of the target sequence provided listeners with a cue to the frequency of the target and decreased informational masking. Ten different maskers were generated for each of the six target frequencies. These same 60 masker sequences were used in the masker-only, uncued-target-plus-masker, and cued-target-plus-masker conditions.

*Experiment 2*: Listeners were presented with target-plus-masker and masker-alone sequences similar to those used in the previous experiment. However, in this experiment, the target and masker tones were presented to the left ear only. Also, all tones were sinusoidally modulated in amplitude (AM depth = 100%). For the target tones, an AM rate of 40 Hz was used to allow recording of the auditory 40-Hz SSR. For the masker tones, the AM rate was randomized between 20 and 50 Hz to maintain perceptual similarity, while avoiding interference between the target and masker evoked SSR [26].

Target tone duration was 250 ms, yielding ten modulation cycles per tone. The target tones were repeated at a constant SOA of 800 ms. The six target-tone frequencies were restricted to the range of 699 to 1,710 Hz (frequency bands 7–12, see also experiment 1). The level of the masker tones was set 6 dB higher than that of the target tones. The masker-tone SOA varied between 250 and 1,350 ms (average SOA = 800 ms).

In their right ear, listeners were presented with a sequence of 100-ms AM tones (10-ms on and off ramps, AM rate = 100 Hz), the frequency of which was randomized between 700 and 1,700 Hz, and the SOA between 250 and 1,350 ms (average SOA = 800 ms). The AM depth was 100% for the standards and 18 dB less for the 10% deviants, which were randomly interspersed among standards. The tone sequence in the right ear continued through the 1.6-s silent gaps separating consecutive stimulus sequences in the left ear.

In a first phase, listeners were instructed to ignore the sounds in their left ear, attend to the sounds in their right ear, and press the right mouse button whenever (and as precisely as possible after) they detected a deviant in that ear. They were not informed that the stimuli presented in their left ear would sometimes contain repeating tones. In a second phase, listeners were instructed to ignore the sounds in their right ear, and to indicate the presence of the target sequence in their left ear, as in experiment 1. In a final phase, the repeating target tones were presented to the listeners' left ear without any masker, while the listeners received the same instructions as in phase 1 (attend right, ignore left).

### Data acquisition.

The MEG was recorded with a Neuromag-122 whole-head system. Data were recorded continuously with a 1,000-Hz sampling rate, in direct coupled mode. Stimuli were presented via foam ear pieces connected to ER-3 earphones (Etymotic research) by 1-m-long plastic tubes. Structural MRI scans (T1 weighted, MPRAGE, voxel size 1 × 1 × 1.3 mm) were obtained from each listener with a 3-T (Siemens Trio) whole-body scanner.

### Data analysis.

MEG activity was averaged relative to the target tones. For sequences containing only maskers, MEG activity was averaged relative to times at which the target tones occurred in the combined sequences. Because the onset times of the masker tones were randomized independently from those of masker tones at other frequencies and from those of the target tones, the activity evoked by the masker tones canceled out in the averaging. In experiment 2, the right-ear task (phase 1 and 3) caused a low-frequency baseline fluctuation that was apparent during the masker-alone condition. Therefore, the average response to masker-alone sequences was subtracted from the average response to the other conditions in experiment 2. In experiments and conditions where listeners indicated when they heard the targets, MEG activity recorded during epochs where listeners had detected the target tones were averaged separately from epochs where the listeners had not detected the target tones. Assuming that the target tones could only be identified after at least two such tones had been heard, the two tones prior to the response were also considered detected. Spatio-temporal dipole source analysis [19] was performed on the averaged data using brain electrical source analysis (BESA). The data were low-pass filtered at 20 Hz (6 dB, zero-phase shift Butterworth filter). A baseline was set 25 ms prior to sound onset, and drifts and slow activity in the subsequent baseline epoch were removed by PCA-based spatial filtering. Dipole analysis of the detected condition was performed in a 100-ms analysis window encompassing the ARN peak. Two dipoles, one in each AC, were fitted to the data. This dipole model was then used as a spatial filter to explore the activation of the AC in the other conditions. For analysis of the 40-Hz SSR in experiment 2, the data were band-pass filtered between 28 and 48 Hz (6 and 12 dB/octave, zero-phase Butterworth filter). The dipole model was fitted to the SSR of the unmasked targets from the control run. These dipoles were used as a fixed spatial filter to generate source waveforms of the SSR for conditions where the masker was present.

The combined data of experiment 1 A and B were also averaged selectively for (a) each of the 12 target presentations, and (b) for each of the six target frequency bands used. Source waveforms were derived with the same dipole model as used for the above analysis (additional 1-Hz high pass filter).

Amplitudes and latencies were measured in the individual source waveforms. ARN and N_1_m amplitudes were measured as the average in the time window 75–175 ms, unless mentioned otherwise. Latencies were measured at the maximum in the time interval 75–275 ms. The peak-to-peak amplitude of the 40-Hz steady-state response was measured after averaging over the ten 25-ms cycles of modulation contained in the 250-ms target-tone duration. Confidence intervals for source waveforms were estimated by calculating *t*-intervals based on standard errors derived with the bootstrap technique based on 1,000 resamples [57]. Dipole positions were co-registered to the individual MRI morphology, and transformed into Talairach space using Brain Voyager.

## Supporting Information

Audio S1 Example of a Target Embedded in an Informational Masker Audio example of a target sequence combined with an informational masker. Listen to these example over headphones in a quiet room. The stimulus is taken from experiment 1B (1,000-Hz target frequency, presented 18 dB below the level of the masker tones; SOA of target and average SOA within each masker stream = 800 ms). You may or may not hear the slowly but regularly repeating target sequence on your first attempt. If you don't hear out the target stream, try listening to the target alone (Audio S2), and, if necessary, adjust volume to an audible, but low and comfortable level, and then return to this example.
(236 KB MP3)

Audio S2 The Isolated Target of the Example Audio S1
Audio example of the target sequence alone. This target is buried among the random informational masker tones of example Audio S1.
(236 KB MP3)

Audio S3 The Isolated Informational Masker of the Example Audio S1
Audio example of the informational masker from example Audio S1 without the target tones. This represents the “catch trials” from the experiment, in which no target was present.
(236 KB MP3)

Figure S1 Detectability (*d'*) of Targets in Multitone Masking The detectability, *d'*, was calculated based on the behavioral data of (A) experiment 1A (200-ms SOA) and 1B (800-ms SOA) and (B) experiment 2 (phase 2). In all three conditions, *d'* increases over the first 4 s and remains relatively stable thereafter. In this context, *d'* is a measure of the amount of informational masking. There was no significant difference between the two SOA conditions in experiment 1. Experiment 2 produced overall a higher amount of informational masking in comparison to experiment 1.
(522 KB PDF)

## Abbreviations

AC: auditory cortex
AM: amplitude modulation
ARN: awareness related negativity
MEG: magnetoencephalography
MRI: magnetic resonance imaging
SOA: stimulus-onset asynchrony
SSR: steady-state response
